# Extracellular Vesicles of Mesenchymal Stromal Cells Can be Taken Up by Microglial Cells and Partially Prevent the Stimulation Induced by β-amyloid

**DOI:** 10.1007/s12015-021-10261-4

**Published:** 2022-01-26

**Authors:** Dorota Kaniowska, Kerstin Wenk, Phil Rademacher, Ronald Weiss, Claire Fabian, Isabell Schulz, Max Guthardt, Franziska Lange, Sebastian Greiser, Matthias Schmidt, Ulf-Dietrich Braumann, Frank Emmrich, Ulrike Koehl, Yarúa Jaimes

**Affiliations:** 1 Fraunhofer Institute for Cell Therapy and Immunology (IZI), Perlickstrasse 1, 04103 Leipzig, Germany; 2 Institute for Clinical Immunology, University of Leipzig, Leipzig, Germany; 3 Department of Isotope Biogeochemistry, Helmholtz Centre for Environmental Research (UFZ), Leipzig, Germany; 4 Faculty of Engineering, Leipzig University of Applied Sciences (HTWK), Leipzig, Germany; 5 Institute for Medical Informatics, Statistics and Epidemiology, University of Leipzig, Leipzig, Germany; 6 Institute of Cellular Therapeutics, Hannover Medical School, Hannover, Germany; 7 Fraunhofer Cluster of Excellence for Immune-mediated Diseases CIMD, Frankfurt, Germany

**Keywords:** Neuroinflammation, Amyloid beta, Alzheimer Disease, Microglia, Mesenchymal stromal/stem cells, Extracellular vesicles

## Abstract

Mesenchymal stromal/stem cells (MSCs) have great capacity for immune regulation. MSCs provide protective paracrine effects, which are partially exerted by extracellular vesicles (EVs). It has been reported that MSCs-derived EVs (MSC-EVs) contain soluble factors, such as cytokines, chemokines, growth factors and even microRNAs, which confer them similar anti-inflammatory and regenerative effects to MSCs. Moreover, MSCs modulate microglia activation through a dual mechanism of action that relies both on cell contact and secreted factors. Microglia cells are the central nervous system immune cells and the main mediators of the inflammation leading to neurodegenerative disorders. Here, we investigated whether MSC-EVs affect the activation of microglia cells by β-amyloid aggregates. We show that the presence of MSC-EVs can prevent the upregulation of pro-inflammatory mediators such as tumor necrosis factor (TNF)-α and nitric oxide (NO). Both are up-regulated in neurodegenerative diseases representing chronic inflammation, as in Alzheimer’s disease. We demonstrate that MSC-EVs are internalized by the microglia cells. Further, our study supports the use of MSC-EVs as a promising therapeutic tool to treat neuroinflammatory diseases.

Significance Statement

It has been reported that mesenchymal stromal/stem cells and MSC-derived small extracellular vesicles have therapeutic effects in the treatment of various degenerative and inflammatory diseases. Extracellular vesicles are loaded with proteins, lipids and RNA and act as intercellular communication mediators. Here we show that extracellular vesicles can be taken up by murine microglial cells. In addition, they partially reduce the activation of microglial cells against β-amyloid aggregates. This inhibition of microglia activation may present an effective strategy for the control/therapy of neurodegenerative diseases such as Alzheimer’s disease.

**Graphic Abstract:**

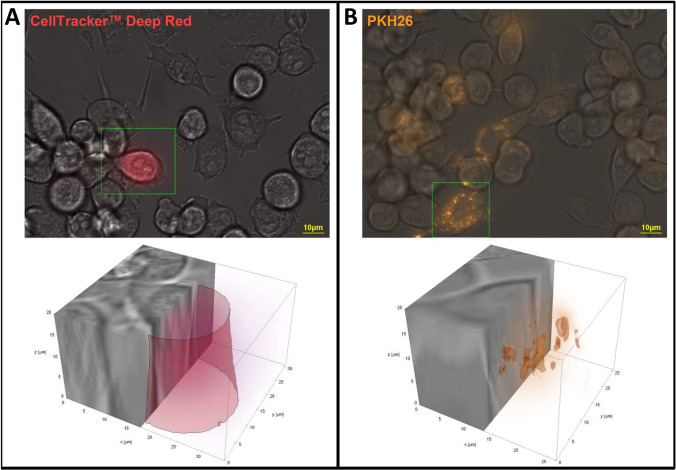

**Supplementary Information:**

The online version contains supplementary material available at 10.1007/s12015-021-10261-4.

## Introduction

Neuroinflammation is a major contributor to Alzheimer’s disease (AD) progression. Brain tissue of patients with AD exhibits chronic inflammation, which is attributed to the activation of microglia cells. The primary role of microglia in the healthy brain is to survey the nervous tissue environment. Despite that, microglia are very reactive cells, their activation results in the overproduction of pro-inflammatory mediators and neuro-toxic cytokines [1–3]. Additionally, activated microglia are commonly associated with amyloid – β (Aβ) containing plaques [4] which can directly stimulate microglia cells to acquire a pro-inflammatory phenotype [5]. In this study, we used Aβ aggregates as stimulus to induce inflammatory responses in the mouse microglia cell line BV-2 as model system since the production of pro-inflammatory mediators by microglia is significantly elevated after Aβ aggregates treatment [6]. Several studies indicated that inhibition of microglial activation provides an effective strategy for the control/therapy of many injuries and diseases, including stroke, multiple sclerosis, neurodegenerative diseases, along with brain trauma [7].

In the last decade, mesenchymal stromal/stem cells (MSCs) have received special interest in the treatment of inflammatory medical conditions, including AD [8–10]. MSCs support their niches *in vivo* by nurturing and promoting the proliferation and differentiation of surrounding cells. Moreover, when transplanted, these cells migrate to sites of inflammation and injury promoting immunomodulation and tissue repair [11]. An easily accessible MSC source with excellent proliferation and differentiation capacity [12, 13], low immunogenicity [14, 15], and ability for immunomodulation [16–20] are adipose stromal/stem cells (ASCs). Injection of ASCs into the cerebral cortex of AD mice led to recovery of spatial learning and memory [21], and intravenous injection of ASCs mitigate dementia in AD mice [8, 22]. The supernatant of ASC cultures is enriched with extracellular vesicles (EVs) which mimic their immune regulatory and regenerative action.

EVs are a heterogeneous population loaded with proteins, lipids and RNA bound by a phospholipid bilayer and functioning as intercellular communication mediators [23]. EVs of MSCs (MSC-EVs) have been reported to display therapeutic effects on the treatment of various degenerative and inflammatory diseases [24–28]. We recently showed that mouse MSC-EVs possess the ability to prevent microglia activation through the modulation of inflammatory cytokines [29]. In order to evaluate different aspects of MSC-EVs in neuroinflammation, we investigated the uptake of human ASC-EVs by BV-2 cells and their capacity to modulate the pro-inflammatory responses of BV-2 cells stimulated with Aβ aggregates.

## Materials and Methods

### Culture of Human ASCs From Adipose Tissue

ASCs were cultured in Dulbecco’s modified Eagle’s medium, 1 g/L D-glucose and GlutaMAX (DMEM-Low Glucose [LG], Life Technologies) supplemented with 20 % FBS (Life Technologies). Medium was changed every 3–4 days. For subculture, cells were passaged weekly with 0.05 % trypsin/ 4 mM EDTA (Life Technologies) and seeded at 3–4.5 × 10^3^ cells per cm^2^ in T175 flasks.

### Flow Cytometry

Surface marker expression was analyzed by flow cytometry. ASCs were used from 4 donors. Cells were incubated at for 4 °C for 15 min in the dark with the following antibodies: CD45 (1:50, REA747), CD73 (1:50, REA804), CD90 (1:50, REA897), CD105 (1:50, REA794) as well as the isotype control (1:50, REA293) (all from Miltenyi Biotec, Germany). Flow cytometry measurement was done on a MACSQuant10 (Miltenyi Biotech) and data was analyzed using FlowLogic (Miltenyi Biotech). A minimum of 10,000 events was analyzed for each sample.

### C57BL/6 Mouse Mesenchymal Stem Cells

Mouse bone-marrow MSCs were purchased from Cyagen US Inc. (Santa Clara, USA). MSCs were cultured in MEM-alpha medium with GlutaMAX, supplemented with 15 % FBS (Life Technologies), 1 % penicillin-streptomycin (Life Technologies) and 2 ng/ml recombinant murine basic fibroblast growth factor (rMu bFGF, Peprotech), with medium changes every three days. The company provides the identity of the cells. Mouse MSCs from Cyagen are positive for CD34, CD44, Sca-1 and negative for CD117. Also, they present multipotential to differentiate towards osteogenic, chondrogenic and adipogenic lineages.

### Extracellular Vesicles Isolation

MSCs culture-supernatant containing the EVs was collected every 72 h starting at passage 2 up until passage 8, at an optical cell confluency higher than 90 %. The EVs were isolated using differential (ultra)-centrifugation steps as previously described [29] (Supplementary Fig. 1). Briefly, cell culture supernatant was centrifuged at 500 x g for 10 min to remove cells and cell debris (Hettich centrifuge, MA). To deplete large vesicles, samples were centrifuged again at 10,000 x g during 30 min and the final concentration of EVs was achieved by centrifuging the supernatant at 70,000 x g during 90 min (Thermo Scientific High-Speed Centrifuge, Germany). All centrifugation steps were performed at 4 °C. The supernatant was discarded via aspiration. EV pellet was resuspended in 0.9 % NaCl or BV-2 cell culture medium and stored at -80 °C, depending on the application. Quantification of EVs was performed by flow cytometry or NanoSight nanoparticle tracking analysis (NTA).

### Extracellular Vesicles Quantification Using Flow Cytometry

The first quantification of EVs was done by FACS Canto II flow cytometer (BD Biosciences) with beads (0.2 and 1 μm) as standard. Based on this quantification, 8 EVs per BV-2 cell were used for the initial experiments. Since quantification by flow cytometry can only be done between 0.2 and 1 μm, later quantification was done using NTA.

### Extracellular Vesicles Quantification and Size Evaluation Using NTA

To evaluate the size distribution and quantity of the EVs in more detail, the samples were evaluated using nanoparticle tracking analysis (NTA, NanoSight LM10, Malvern Panalytical, Software “NTA 3.0”). All samples were diluted 40 folds in 0.9 % NaCl to a final volume of 1 ml. The ideal measurement concentrations were found by pre-testing the optimal particle per frame value (20–100 particles/frame). Following settings were set according to the manufacturer’s software manual (NanoSight NS300 User Manual, MAN0541-01-EN-00, 2017): camera (type SCMOS) level was increased until 8 so that all particles were distinctly visible, slide shutter 350, slide gain 250, and temperature 25 °C.The software NTA 3.0 was used for the analysis of three videos of 60 s recorded per sample, with a detection threshold of 5.

### Scanning Helium-ion Microscopy Examination of ASC-EVs

EVs isolated from the culture medium of ASCs were morphologically evaluated using scanning helium-ion microscopy. The ASC-EVs were fixed in 2.5 % glutaraldehyde in PBS, pH 7.4 and incubated at 4 °C at least for 24 h. Later, the vesicles were slowly filtered onto a ceramic filter with a pore size of 20 nm (GE Healthcare, Sweden) supported by a mixed cellulose ester (MCE) membrane filter with 0.45 μm pore size in a manual filtration unit (Sartorius). Still in the filtration unit, the ASC-EVs on the filter were washed with 500 µl of PBS. Subsequently, the filter was removed and dehydrated in a graded ethanol-series starting with 30 % ethanol in a dilution series up to absolute ethanol. Then the filter was critical point dried (CPD) using a Leica CPD300 critical point drier (Leica Microsystems, Germany) in order to make them vacuum-compatible. During 20 CPD-cycles the ethanol was exchanged by super-critical CO_2_, which afterwards evaporated. After the drying process, a part of the ceramic filter was glued onto an aluminum-specimen stub (Agar Scientific, UK) using air-drying conductive silver epoxy (Acheson 1415 Plano, Germany). Then, the samples were sputter-coated with a conductive layer of gold-palladium (9:1) of 30 nm thickness using a tabletop sputtering device Leica EM SCD 500. The micrographs were acquired with a Zeiss Orion NanoFab HIM (Carl Zeiss Microscopy, MA). He^+^ ions with an ion-landing energy of 25 kV and a beam current of about 0.7 pA allowed for high surface sensitivity and minimum damaging of the specimen. Secondary electrons were detected using an Everhart-Thornley-type secondary electron detector for imaging, with a pixel dwell time of 0.5 µs. Noise reduction was achieved by scanning and averaging each line for 64 times.

### Multiplex Analysis of ASC-EV Surface Markers

The presence of vesicle surface markers in ASC-EVs was analyzed using the MACSPlex exosome, human, Kit (Miltenyi Biotec) following the manufacturer’s instructions. Briefly, 50 µl of the antibody-coated capture beads targeting thirty-seven different membrane antigens were incubated during 16 h with 1.5 × 10^9^ ASC-EVs resuspended in 120 µl of 0.9 % NaCl. Later, an antibody cocktail targeting the tetraspanins CD9, CD81 and CD63 was added and incubated for 1 h. The flow cytometry analyses were done using the FACS Canto II (BD Biosciences) and the FACS Diva software (BD Biosciences). The calibration of the flow cytometer was set as recommended by the kit manufacturer using the setup beads (Singlets) provided. The values of median fluorescence intensity below the provided corresponding control antibodies were considered as threshold and as negative. Data are normalized to the mean values of the median fluorescence intensities of the tetraspanins (CD9, CD63 and CD81). The samples were evaluated in three independent runs.

### BV-2 Stimulation with Β-amyloid Aggregates

The immunomodulatory capacity of human ASC-EVs or mouse MSC-EVs in the prevention of the microglia cells activation towards stimulation with Aβ aggregates was tested *in vitro*. BV-2 cells were seeded at 2.1 × 10^5^ cells/cm^2^ in a 24-well plate and incubated 24 h in DMEM-HG with GlutaMAX and 2 % FBS (Life technologies). Microglia cells were primed using 1 µg/ml of lipopolysaccharides (LPS, Sigma-Aldrich) for 3 h. Later, the cells were washed with cell culture media, prior stimulation with 10 µM of Aβ aggregates for 6 h (for evaluation of gene expression) or 24 h (for evaluation of cytokine secretion and nitric oxide (NO) production) at 37 °C and 8 % of CO_2_. Unstimulated cells were used as negative control (NC). Eight human ASC-EVs or murine MSC-EVs per cell (flow cytometry quantification) were added to BV-2 cells in the respective wells. As control for the effect of the vesicles on BV-2 cells, the cells were incubated with the vesicles after priming and with no further stimulation.

### Live Cell Imaging of EV/microglia Co-culture

Immediately after adding the EVs stained with PKH26 Red Fluorescent Cell Linker or CellTracker™ Deep Red dye to BV-2 cells, live cell imaging was performed using an inverse microscope ZEISS Axio Observer.Z1 (Carl Zeiss AG, Germany) equipped with AxioCam MR Rev3 camera (image size of 1388 × 1040 pixel, bit depth of 12), Plan-Apochromat 63x/1.40 oil objective and live cell incubation setup consisting of Heating Unit XL at temperature of 37 °C and CO_2_ Module S at 8 % CO_2_ (PeCon GmbH, Germany). Measurements were done in z-stacks with Brightfield and Fluorescence exposure time of each 300 ms, filter set no. 50 (Carl Zeiss AG) for Cy5, excitation filter at 625–655 nm, emission filter at 665–715 nm and dichroic mirror at 660 nm for EVs with CellTracker™ Deep Red dye as well as brightfield exposure time of 80 ms, fluorescence exposure time of 220 ms, filter set no. 43 (Carl Zeiss AG) for DsRed, excitation filter at 538–562 nm, emission filter at 570–640 nm and dichroic mirror at 570 nm for EVs with PKH26 Red Fluorescent Cell Linker dye.

### Statistical Analysis

Each experiment was performed at least three independent times. The data represents the mean ± SEM calculated from all assays. Statistical significance was calculated using the Mann Whitney U-Test or Student´s *t*-test as indicated and shown with asterisks (*p < 0.05, **p < 0.01, ***p < 0.001). All analyses were done with the Graph Pad Prism v.6.02 (San Diego, CA, USA) or SigmaPlot 14.0 (Systat Software) software.

## Results

### Human ASCs From Donors of Different Ages Maintain MSC Characteristics

**Fig. 1:**
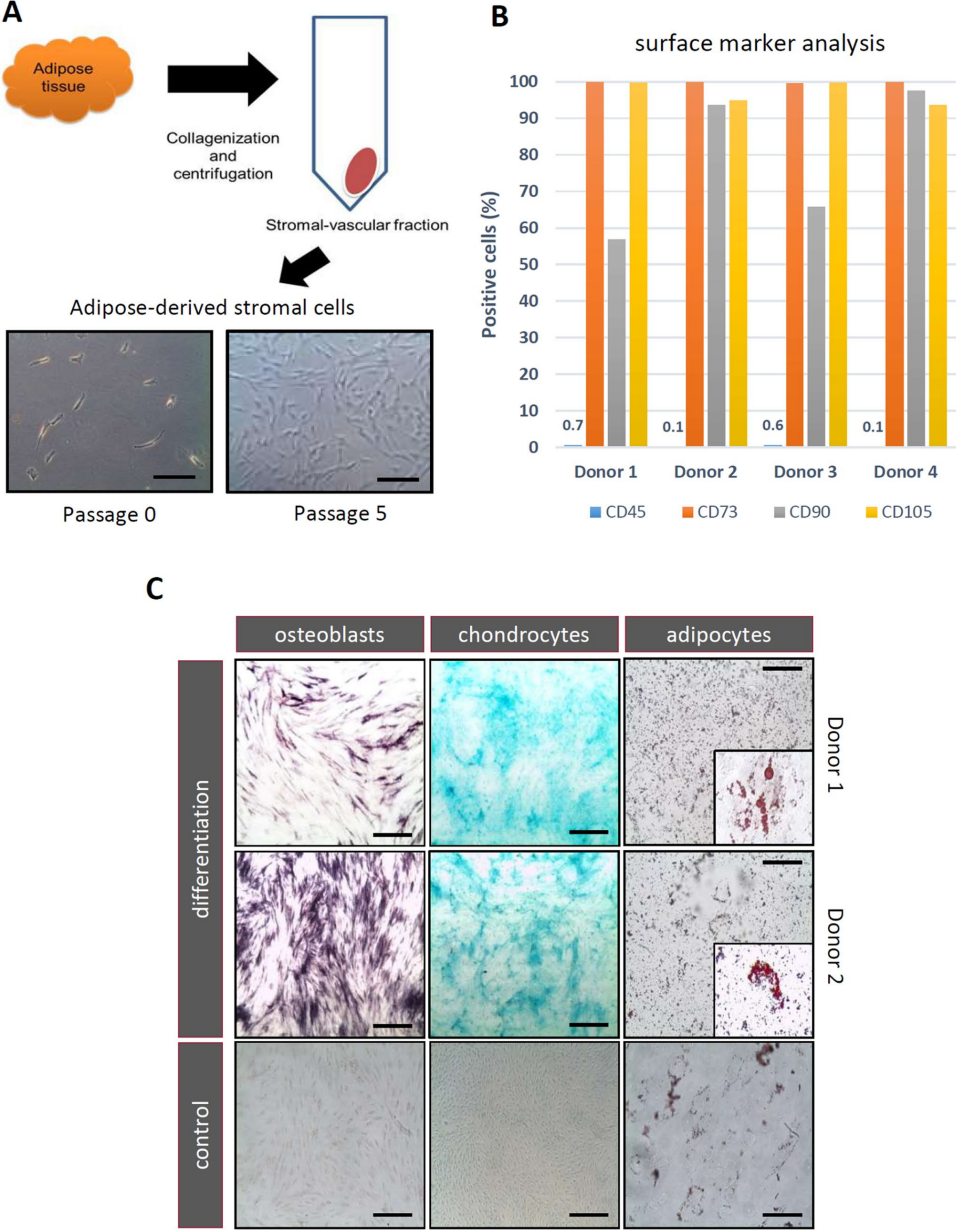
Isolation and characterization of human adipose derived cells (ASCs). (**A**) Human subcutaneous adipose tissues (2–10 gr) from different age groups and donors were digested with collagenase IV. After centrifugation, the stromal-vascular fraction was isolated and transferred to flasks completely filled with culture medium. The ASCs were cultured in 20 % FBS DMEM-LG with medium changes every 3–4 days. The cell surface expression of the common ASC markers was evaluated by flow cytometry of 4 immunomodulatory donors (**B**), showing that the cells did not express hematopoietic lineage markers, such as CD45, and were positive for CD73, CD90 and CD105 n = 4. (**C**) Cytochemical staining of differentiated and undifferentiated ASCs. Fast Red staining after osteogenic differentiation show a calcium deposition. Alcian blue staining after chondrogenic differentiation show marked deposition of glycosaminoglycans in the matrix. Oil red O stain after induction of adipogenic differentiation show cytoplasmic neutral triglyceride droplets. Scale bars = 100 μm

The adipose-derived stromal vascular fraction (SVF) is a heterogeneous population of cells including MSC-like cells, endothelial progenitor cells, and hematopoietic cells. ASCs were isolated from adipose tissue obtained from eyelid and breast (Fig. 1A) and analyzed according to the position statement of the International Society for Cellular Therapy [30, 31]. The ASC yield was much greater for donors aged less than 40 years compared to older donors; the yield gradually fell as the donor age rose (data not shown). The human ASCs have grown *in vitro* as typical spindle-like structures adherent on tissue culture plastic without obvious changes in shape, size or density (Fig. 1A). The surface protein expression of human ASCs at early passage was examined by flow cytometry (Fig. 1B). ASCs were positive for stem cell markers CD73, CD90, and CD105. Conversely, isolated ASCs had a low or no expression of the hematopoietic marker CD45. There was no difference between groups regarding cell morphology (data not shown). ASCs were also evaluated in terms of their multilineage capacity to exhibit chondrogenic, osteogenic and adipogenic differentiation (Fig. 1C). Three-lineage differentiation assay was performed to test the multipotency of human ASCs. After two-week adipogenic differentiation, massive amounts of lucent lipid droplets of a high refractivity could be visualized in the cytoplasm as observed under a microscope, and the color red was developed after using Oil Red O. The osteogenically-differentiated cells in the monolayer culture were stained by Fast red and proteoglycan-rich cartilaginous matrix produced during chondrogenic differentiation were visualized by Alcian blue. These results indicated that we obtained human ASCs with high purity and multipotent ability.

### Screening the ASC-EVs Immunomodulatory Capacity

**Fig. 2:**
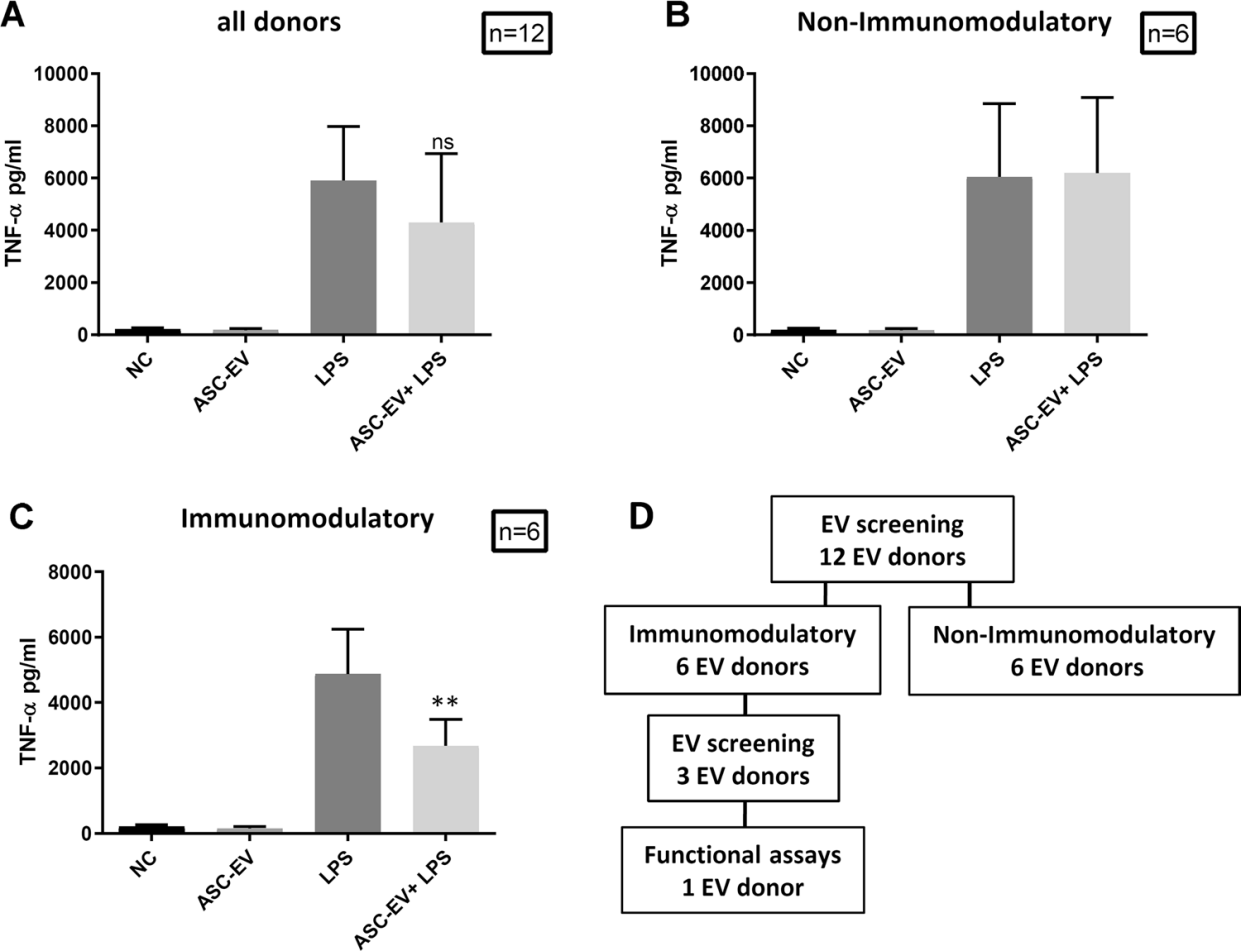
Immunomodulatory screening of extracellular vesicles from adipose stromal/stem cells (ASCs) of different donors. EVs from the ASC supernatant were isolated via differential (ultra)-centrifugation. Resting BV-2 cells were primed for 3 h with 1 µg/ml LPS before addition of 8 EVs/cell (quantification by flow cytometry) for 24 h. The secretion of TNF-α was evaluated by ELISA in the cell-culture supernatant as an activation read-out for the LPS-stimulated microglia cells. (**A**) The TNF-α levels from all donors (n = 12) were compared. The donors were separated into (**B**) non-immunomodulatory (n = 6) and (**C**) immunomodulatory donors (n = 6). Statistics were calculated with the Mann Whitney U-Test. NC = negative control (**D**) Further characterization and functional analysis of EVs were carried out for the immunomodulatory EVs as shown in the scheme

Previously we demonstrated the immunomodulatory capacity of mouse MSC-EVs towards LPS stimulated BV-2 cells [29]. In order to assess whether human ASC-EVs act as BV-2 immune modulators in a comparable way to mouse MSC-EVs, we used the same LPS stimulation assay of BV-2 cells and measured the TNF-α secretion using ELISA. EVs from the cell culture supernatants were isolated via differential (ultra)-centrifugation [29] (Supplementary Fig. 1). The capacity of human ASC-EVs to prevent TNF-α secretion by LPS stimulated BV-2 cells was highly variable among all donors (Fig. 2A; n = 12). However, the half of the tested donors (n = 6) showed no effect on the secretion of TNF-α, regardless of the concentration, whereas the other half (n = 6) showed a strong immunomodulatory potential (Fig. 2B, C). Despite different functional activities of the ASC-EVs, CD73 was expressed equally among all donor ASCs (data not shown). We selected the immunomodulatory donors for EV characterization and one donor for testing the effect on BV-2 cells after stimulation with Aβ aggregates (Fig. 2D).

### Characterization of ASC-derived EVs

**Fig. 3:**
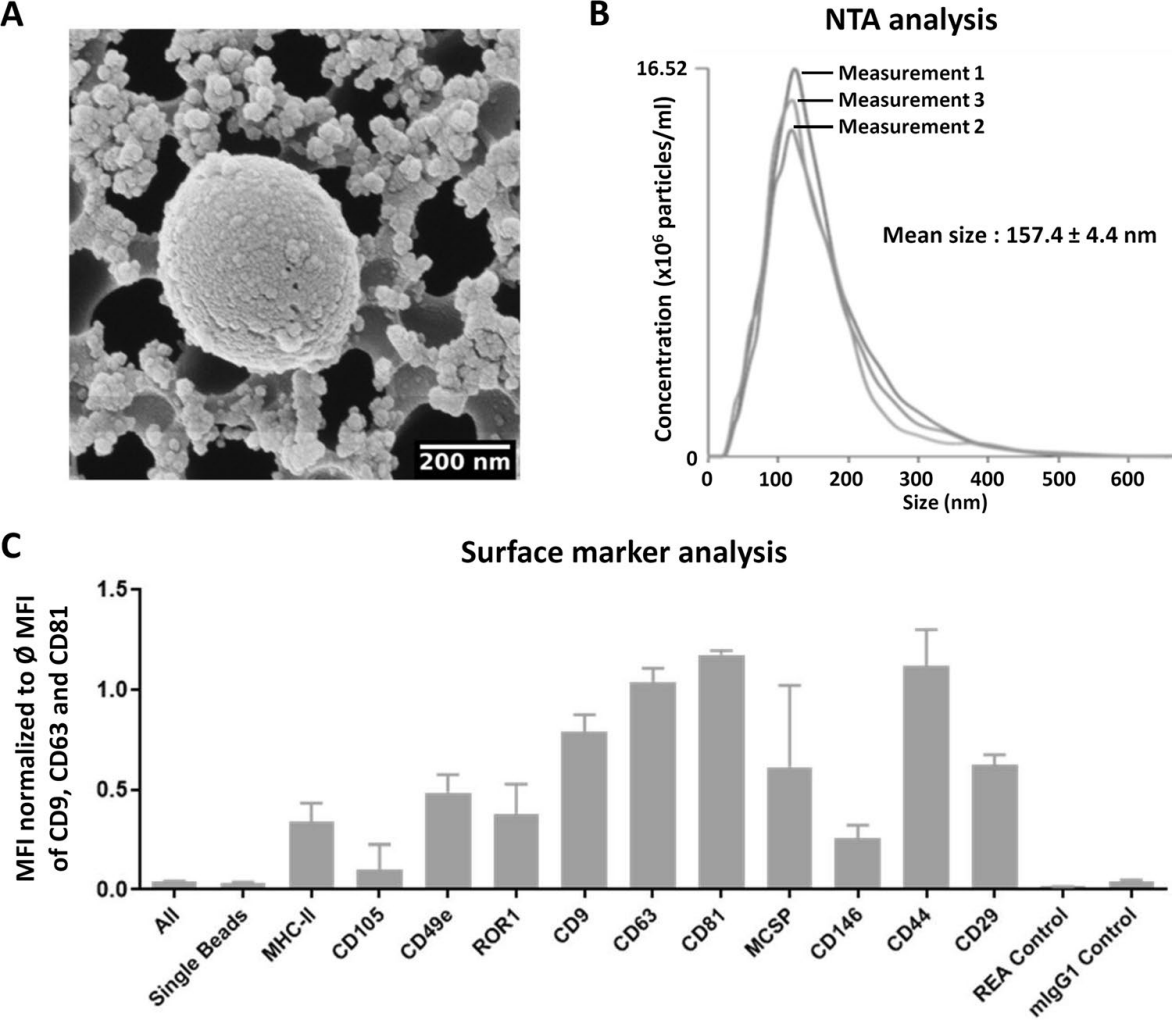
Characterization of EVs isolated from adipose stromal/stem cells (ASCs) culture supernatant. EVs from the ASC supernatant were isolated via differential (ultra)-centrifugation. (**A**) One representative micrograph of EVs isolated from ASCs taken by scanning helium-ion microscopy. (**B**) One representative graph of ASC-EV size evaluation using NanoSight nanoparticle tracking (NTA) analysis. ASC-EVs isolated from cell culture supernatant show a mean size of 157 nm. (**C**) MACSPlex analysis of the ASC-EV surface markers

Human ASC-derived EVs were characterized according to the minimal information for studies of EVs [32] using helium ion microscopy for structural evaluation and visualization, NanoSight for quantification and size evaluation, and MACSPlex assays to evaluate the present surface antigens. Although EV biogenesis, cargo, and function might widely differ, EVs remain mainly classified according to their size unless specific markers for subcellular origin are established. Using helium ion microscopy, the ASC-EVs were visualized as spherical structures with sizes ranging from small EVs (~ 30 nm) to medium/large EVs (~ 400 nm), confirming herewith the heterogeneity of the EV population (Fig. 3A). The NanoSight evaluation also showed that the human ASC-EVs had dimensions ranging from 30 to 400 nm with a mean of about 157 nm and a particle concentration of 3.1 × 10^8^ particles/ml of original cell culture supernatant (Fig. 3B). In addition, using flow cytometry, we analyzed the surface expression of various molecules on human ASC-EVs (Fig. 3C). We detected 11 different molecules variably expressed; in particular, the tetraspanins and genuine EV markers CD9, CD63 and CD81. Moreover, EVs released from human ASCs were positive for the MSC markers: CD29, CD44, CD105 and CD146 and negative for CD45 (data not shown). Besides, a higher expression of MCSP and ROR1, cancer markers, with strong median fluorescence intensities (MFI) were found.

### Stimulation of BV-2 Cells with Aβ Aggregates

**Fig. 4:**
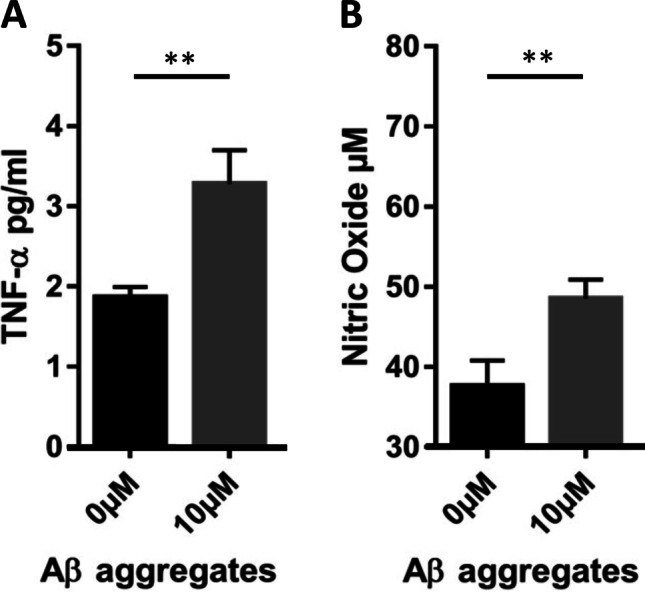
Activation of BV-2 cells with Aβ aggregates. Resting BV-2 cells were primed for 3 h with 1 µg/ml LPS before stimulation with 10 µM of Aβ aggregates for 24 h. The cell culture supernatants were finally analyzed for secretion of (**A**) TNF-α by ELISA and (**B**) NO using a Griess reagent. Data represent means ± SEM (n = 3). Statistics were calculated with the Student´s *t*-test

Neuroinflammation in AD is associated with an increase of the number of microglia, accompanied by phenotypic changes leading to an enhanced cytokine and chemokine release. Aβ aggregates stimulate microglia cells to produce pro-inflammatory signals, which are neurotoxic mediators and might propagate an inflammatory cycle in the central nervous system (CNS) [5]. In order to establish an *in vitro* model for microglia stimulation by Aβ aggregates, we used a widely reported protocol for Aβ (1–42) aggregation during 86 h at 37 °C in PBS [33, 34]. Resting BV-2 cells did not show to be responsive towards Aβ aggregates without prior stimulation (data not shown). Hence, BV-2 cells were primed during 3 h with LPS to induce microglia activation. An overview of the experimental procedure is shown in Supplementary Fig. 2. Activated BV-2 cells were stimulated with different concentrations of Aβ aggregates (1 µM, 2.5 µM, 5 µM, 10 µM and 20 µM) and responses were assessed by measuring TNF-α secretion (Supplementary Fig. 3A) and nitric oxide (NO) release (Supplementary Fig. 3B). A clear upregulation of TNF-α (Fig. 4A) and NO (Fig. 4B) was reached at 10 µM of Aβ aggregates, hence that was the concentration selected for all further stimulation experiments.

### Suppression of Pro-inflammatory Molecules by EVs in BV‐2 Cells Stimulated with Aβ Aggregates

We recently showed that EVs derived from mouse MSCs attenuate the production of inflammatory molecules induced by LPS stimulation of BV-2 cells [29]. In accordance with these investigations, we examined the influence of human ASC-EVs and mouse MSC-EVs on cytokine production by Aβ-stimulated mouse BV-2 cells to assess whether EVs of different species performed the same function on mouse BV-2 cells.

**Fig. 5:**
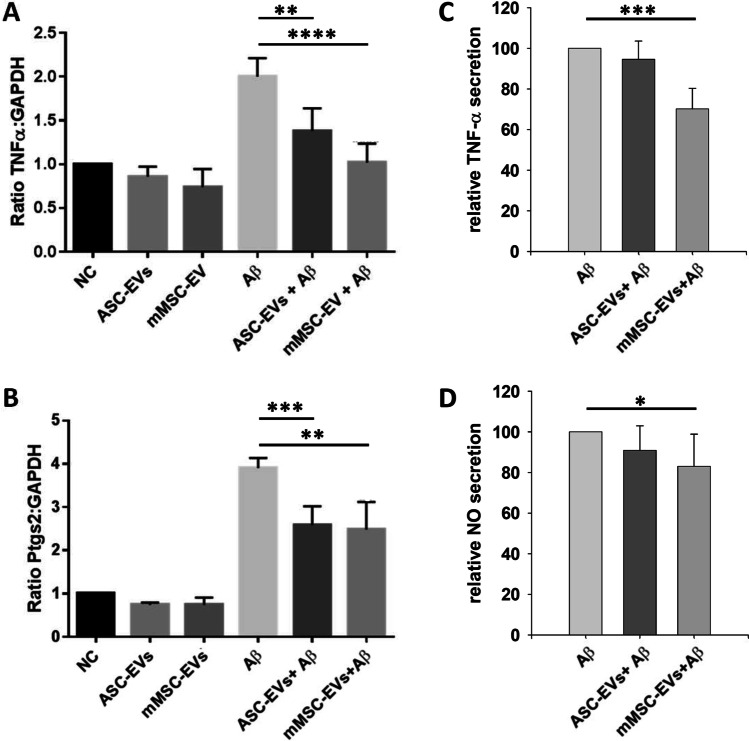
The human ASC-EVs and mouse MSC-EVs treatment partially prevents the upregulation of pro-inflammatory molecules in BV-2 cells after Aβ stimulation. EVs from the human ASC and mouse MSC supernatants were isolated via differential (ultra)-centrifugation. Resting BV-2 cells were primed for 3 h with 1 µg/ml LPS before stimulation with 10 µM of Aβ aggregates in the presence and absence of 8 EVs/cell (flow cytometry quantification). Gene transcription of (**A**) TNF-α and (**B**) PTGS2 were evaluated by RT-PCR 6 h after stimulation. After 24 h of stimulation, secretion of the pro-inflammatory molecules (**C**) TNF-α and (**D**) NO to the cell culture supernatant were evaluated using ELISA and Griess reagent reactions, respectively. Data represent means ± SEM (n = 3). Statistics were calculated with the Mann Whitney U-Test (A and B) or with the Student´s *t*-test (C and D)

Therefore, BV-2 cells were co-cultured with human ASC-EVs or mouse MSC-EVs in the presence and absence of Aβ aggregates. The concentrations of EVs was the same used in our previous study, where we also tested the immunomodulatory potential of mouse MSC-EV on microglia cells [29]. The mRNA expressions of TNF-α and PTGS2 were evaluated 6 h after stimulation, while secretions of TNF-α and NO were analyzed after 24 h. As shown in Fig. 5A and B the stimulation of BV-2 cells with Aβ aggregates induced an upregulation of TNF-α and PTGS2 transcripts, compared to the controls. Importantly, the presence of human ASC-EVs or mouse MSC-EVs significantly prevented the upregulation of these pro-inflammatory molecules. Addition of human ASC-EVs or mouse MSC-EVs to only LPS primed cells did not have any significant effect on the transcription of TNF-α and PTGS-2 (Fig. 5A and B).

However, Aβ-stimulated BV-2 cells showed a two times higher concentration of TNF-α and NO in the cell culture supernatant compared to non-stimulated cells (data not shown). A significant EV effect on Aβ-stimulated BV-2 cells were observed on TNF-α and NO protein levels induced by mouse MSC-EVs. The human ASC-EVs had no effect on TNF-α and NO secretion of Aβ-stimulated mouse BV-2 cells. (Fig. 5C and D)

### The β-amyloid Receptor CD36 is Affected by Human and Mouse MSC-derived EVs

**Fig. 6:**
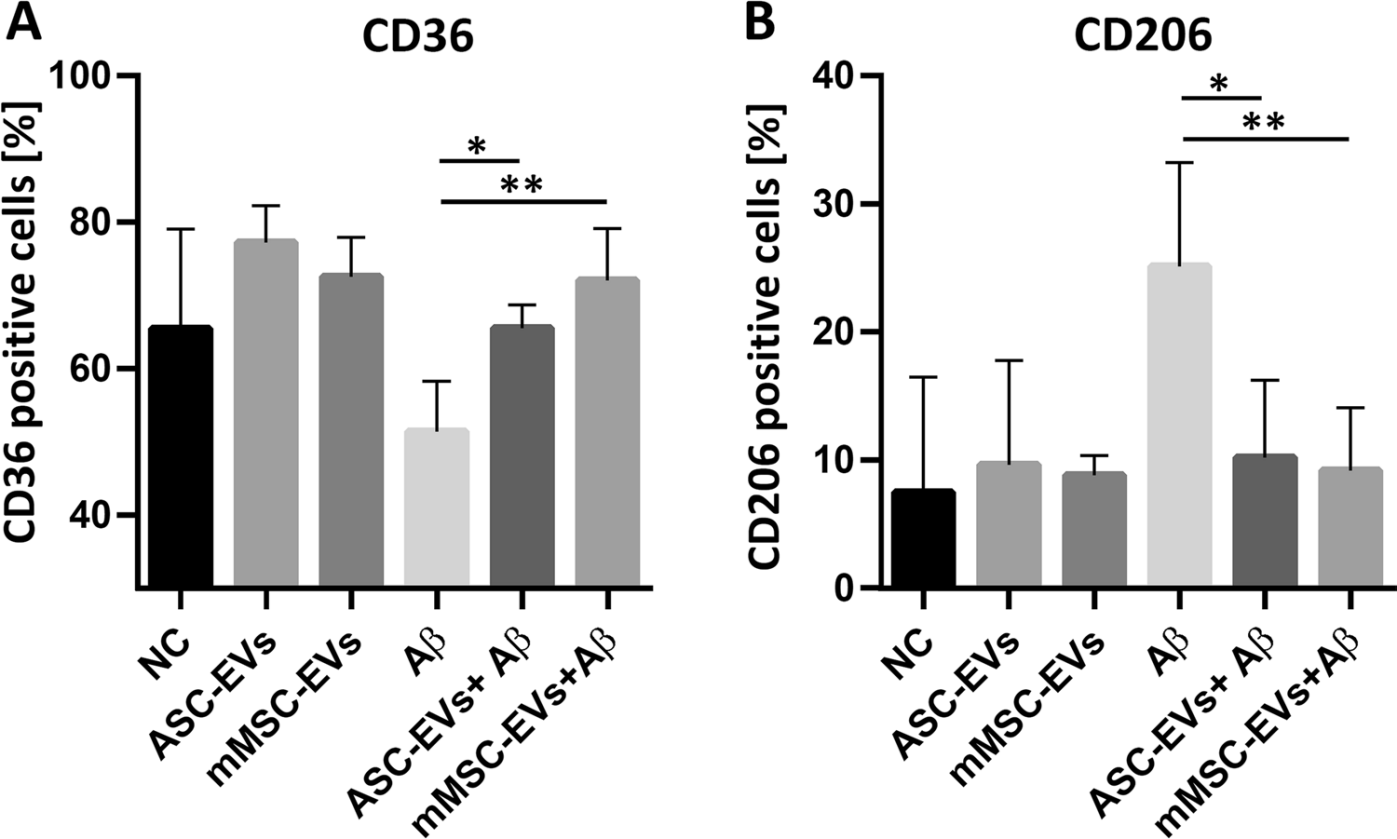
The human ASC-EVs and mouse MSC-EVs affect the expression of CD36 and CD206 of BV-2 cells after Aβ stimulation. EVs from the human ASC and mouse MSC supernatants were isolated via differential (ultra)-centrifugation. Resting BV-2 cells were primed for 3 h with 1 µg/ml LPS before stimulation with 10 µM of Aβ aggregates in the presence and absence of 6.5 × 10^4^ ± 1.5 × 10^4^ EVs/cell (NTA quantification). After 24 h of stimulation surface expressions of (**A**) CD36 and (**B**) CD206 on BV-2 cells were evaluated by flow cytometry. Data represent means ± SEM (n = 3). Statistics were calculated with the Mann Whitney U-Test

The cell surface receptor CD36 is a class B scavenger receptor, expressed on microglia cells in normal brains and brains from AD patients. CD36 mediates macrophages and microglia responses to Aβ aggregates, playing a major role in the pro-inflammatory events associated with AD [35]. Approximately 65 % of BV-2 cells express CD36 after priming (Fig. 6A). We observed that the CD36 expression decreases to approximately 51 % after stimulation with Aβ aggregates during 24 h, which has been described as a common process, due to the internalization of the molecules during phagocytosis [36, 37]. Interestingly, BV-2 cells stimulated with Aβ aggregates in the presence of human ASC-EVs or mouse MSC-EVs show a significant increase of CD36, compared to cells stimulated in absence of the EVs (64 %, *p = 0.0286* and 72 %, *p = 0.0095*, respectively).

### MSC-derived EVs Prevent the Upregulation of CD206 By BV-2 After Β-amyloid Stimulation

CD206 is a C-type lectin and mannose receptor usually used as a surface marker for M2 macrophages [38, 39]. We observed that CD206 is expressed by 7 % of the LPS primed BV-2 cells (NC) (Fig. 6B). While, after stimulation with Aβ aggregates, the number of CD206 expressing cells gained up to 25 %. Interestingly, co-culture with human ASC-EVs or mouse MSC-EVs significantly prevented the upregulation triggered by Aβ stimulation (10 %, *p = 0.0152* and 9 %, *p = 0.0087* respectively). The expression of the cell surface molecules CD45, CD11b, CD86 and CD80 was also evaluated and remained unaffected after BV-2 stimulation with Aβ aggregates (data not shown).

### Human ASC-EVs and Mouse MSC-EVs do not Affect the Uptake of Latex Beads by BV-2 Cells

The internalization of the Aβ complexes occurs via phagocytosis [6]. Given the observation that internalization of CD36 was prevented in presence of human ASC-EVs and mouse MSC-EVs (Fig. 6A), we evaluated the general phagocytic capacity of microglia cells in the presence of EVs. Therefore, we established a phagocytosis assay based on flow cytometry that measures the bulk phase uptake of latex beads to examine the phagocytic response. First, we incubated BV-2 cells with latex beads and found that almost 67 % of all BV-2 cells took up at least one bead. LPS priming of the cells slightly suppressed the phagocytosis of latex beads. However, no statistically significant difference was found in beads phagocytosis between LPS untreated and treated cells (Supplementary Fig. 4). Stimulation of BV-2 cells with Aβ aggregates prevented the latex bead uptake. However, the presence of human ASC-EVs or mouse MSC-EVs did not show any influence on the capacity of BV-2 cells to take up latex beads.

### ASC-EVs are Internalized by Microglia Cells

EVs are expected to have different mechanisms for interacting with the cells. In order to deliver a signal, EVs need to fuse with the membrane of the target cells, either with the plasma membrane or after endocytic uptake with the endosomal membrane [40]. We demonstrated that human ASC-EVs influence the inflammatory responses of stimulated microglia cells (Fig. 5). However, the mechanism of vesicle-cell interaction is still an open question. In order to understand how human ASC-EVs might deliver a signal to BV-2 cells, two different EV labelling protocols were used: (i) the vesicle content stained with CellTracker™ Deep Red dye and (ii) the vesicle membrane stained with PKH26 Red Fluorescent Cell Linker. It was observed utilizing live cell imaging that the vesicles only internalize in a subset of BV-2 cells for both protocols.

Moreover, the content of the vesicles (Fig. 7A), if released, and the vesicle membrane (Fig. 7B) were spread anywhere inside of a BV-2 cell. Based on z-stacks obtained during live cell imaging, and after subsequent 3D image post processing and 3D visualization applying a combination of both surface and volume rendering using Wolfram Mathematica 12 (Wolfram Research Inc., Champaign, IL, USA), we could determine that the vesicle content was dissolved and the membranes of the ASC-EVs seemed to be remaining centrally inside the cytoplasm of the BV-2 cells. This indicates that the mechanism of interaction involves the uptake of the complete vesicle and not only the fusion of the EVs with the cell membrane.

Furthermore, the EV-membranes were exchanged via cell junctions between cells in culture over a timescale of 5 days (Supplementary video 1). Hence, we demonstrate that ASC-EVs are internalized by BV-2 cells and that the vesicle content is released inside the cells.

## Discussion

Neuroinflammation is a major problem in neurodegenerative diseases as Alzheimer disease [41]. The microglia cells, as the CNS immune cells, are in charge of the immune surveillance, and become easily activated towards various stimuli. However, the functionality of microglia cells is important for the homeostasis maintenance. Hence, a tight balance between responsive but not activated microglia cells is essential in order to regulate the chronic inflammation leading to neurodegeneration. In a previous study, we had demonstrated that mouse MSC-EVs represent powerful modulators of mouse BV-2 and primary microglia responses towards LPS stimulation [29]. Here, we show that MSC-EVs can act also as immune modulators of microglia responses after stimulation with Aβ aggregates.

Human microglia cells can be obtained from human brains post mortem only. Therefore, most of the research about the inflammatory responses of microglia cells has been gathered using murine cells, *in vitro* and/or *in vivo*. Hence, assessing new therapeutic approaches for neurodegenerative diseases involving microglia cells represents an enormous challenge. To develop *in vitro* models that mimic the inflammatory processes occurring in the diseased CNS is of major importance for initial screening of possible therapeutic agents. The mouse cell line BV-2 has been repetitively used as an alternative model system for primary microglia cultures or for animal experiments evaluating brain inflammation [42]. Our goal was to assess the immunomodulatory capacity of MSC-EVs towards microglia cells. Recently it was shown, that the MSC source and the culture media conditions notably influence the properties of MSCs and the characteristics of the extracellular vesicles they shed [43]. For our experiments we used adipose tissue of breast and eyelid instead of liposuction because of a better isolation effect and a stronger proliferative and regenerative potential *in vitro* [44, 45]. Furthermore, we used 20 % FBS in the culture medium of the MSCs for a better behavior of the cells. In initial experiments we were able to rule out the effects of FBS-derived EVs compared to MSC-EVs (data not shown). Under the chosen experimental conditions, we were able to demonstrate that EVs derived from human ASCs, exert effects comparable to those from mouse MSCs on modulating the inflammatory responses of microglia cells on mRNA level. Furthermore, our analysis of EVs derived from human ASCs confirmed the concurrent presence of tetraspanins as EV marker and of MSC-derived marker. Importantly, the α5 integrin subunit of the fibronectin receptor (CD49e) was detected on the vesicles. The presence of low levels from this marker on MSCs has been associated with osteoblast differentiation in various studies [46, 47]. In AD, the aggregates that form Aβ plaques are closely associated with activated microglia cells. These cells produce molecules as TNF-α, IL-1β, IL-6 and NO which are known to promote neuroinflammation [48]. The Aβ peptides are metabolism products usually formed by 36 to 43 amino acids. There are several Aβ forms found in the brain, but toxicity to the cells has been demonstrated by the Aβ peptides 1–42 [49]. *In vitro* models to assess the inflammatory responses occurring in the AD brain represent the first approach to evaluate possible therapies. Here, we established an *in vitro* model for microglia stimulation with Aβ 1–42 aggregates [33, 34] in which we can induce the main responses reported in the AD brain for activated microglia cells. Our model allows assessing the production of pro-inflammatory molecules as TNF-α, IL-6 and NO, which are prevalent in chronic neuroinflammation diseases [50]. Interestingly, we did not observe an upregulation of IL-1β expression in microglia cells after stimulation with Aβ 1–42 aggregates (data not shown). Moreover, following our described protocol [29], BV-2 cells require previous priming in order to induce inflammatory responses strong enough for assessing the molecular methods available at our laboratory.

The EVs by themselves do not trigger any inflammatory stimuli; however, their specific content affects the microenvironment. An immunosuppressive capacity of MSC-EVs has been reported previously by Wang et al., showing that human MSC-EVs prevent life-threatening acute Graft Versus Host Disease by modulating immune responses [28]. Subsequently, Sun et al. observed that human MSC-EVs promote functional recovery in spinal cord injury via attenuating inflammation [51]. We could show that both human ASC-EVs and mouse MSC-EVs significantly down-regulated the transcripts of pro-inflammatory mediators as TNF-α and PTGS2. However, only BV-2 cells stimulated with Aβ in presence of mouse MSC-EVs showed a significant lower secretion of TNF-α and NO, indicating less inflammatory and more neuroprotective effects. The observation of neuroprotective effects from MSC-EVs mediated by the prevention of NO secretion was also previously reported [52]. The group reported that MSC-EVs present high catalase activity, which might provide the protective action against neuronal oxidative stress. The human ASC-EVs had no significant effect on secreted TNF-α nor on NO, which may be explained by the species barrier between human ACS-EVs and mouse BV-2 cells. However, we were able to show that EVs can have the potential to modify the inflammatory response of immune cells by using the mouse-derived MSC-EVs on mouse microglia BV-2 cells.

The percentage of BV-2 cells expressing the surface Aβ receptor CD36 decreased after stimulation with Aβ aggregates. This reduction was described to be associated with the Aβ internalization [35]. Interestingly, the presence of human ASC-EVs and mouse MSC-EVs prevented this reduction, which might imply a prevention of Aβ internalization. Due to this observation, we hypothesized that the MSC-EVs could affect the phagocytic capacity of microglia cells. However, we did not observe any significant influence in the capacity of BV-2 cells to internalize latex beads. Further it could be important, to evaluate whether the MSC-EVs directly affect the Aβ internalization. In contrast, upregulation of CD36 and CD206 expression in response to Aβ aggregates was significantly prevented in presence of human and mouse MSC-EVs. The mannose receptor CD206 is commonly associated with the anti-inflammatory activation of macrophages [38, 39], and increased in the late stage of lesions developed by ischemia patients [53]. Taking together, we observed that the autologous setting, using mouse MSC-EVs showed a slightly stronger effect at preventing pro-inflammatory responses of BV-2 cells towards Aβ than using human ASC-EVs.

The interaction mechanism of EVs with the target cell might highly vary [40]. Here, we could demonstrate using live cell microscopy that the human ASC-EVs are internalized by a random subset of BV-2 cells, and the EV-content is distributed in the cytoplasm. The EV-membranes were spread towards neighbored BV-2 cells via tubuli, which suggests that the signals delivered by the EVs might reach more than one cell. Hence, from a therapeutic perspective it should be considered that even the local application of MSC-EVs could provide broader systemic effects. As we showed previously, in BV-2 cells, MSC-EVs prevent the MAPK phosphorylation signaling cascade which occurs after LPS stimulation, therewith leading to lower transcription of genes associated with inflammation [29]. MSC-EVs transport a broad number of bioactive molecules; however, the specific EV delivered molecules that are involved in preventing the inflammatory responses from microglia cells, remain to be elucidated.

## Conclusions

In conclusion, our findings demonstrate that EVs derived from human ASC and mouse MSCs modulate the activation of BV-2 microglia cells and can prevent the pro-inflammatory response. Therefore, ASC-EVs might be a promising tool for new therapeutic approaches targeting chronic inflammation leading to neurodegeneration, including AD. Moreover, we have shown that human ASC-EVs are internalized by microglia cells, and that the content is distributed to neighboring cells. Thus, EVs have the potential to amplify signals which might be used in therapeutic approach. Furthermore, MSCs are very attractive in regenerative medicine due to their potential in tissue protection and regeneration. Thus, our findings provide hints that the mechanism of tissue protection may also be carried out by MSC derived EVs.

## Supplementary Information

ESM 1 Internalization of human ASC-EVs by BV-2 cells.ASC-EVs were labelled with CellTracker™ Deep Red dye, which labels the internal vesicle content (**A**), or with PKH26 Red Fluorescent Cell Linker, which labels the vesicles membranes (**B**), and were than co-cultured with BV-2 cells for up to 5 days. Live cell imaging was performed using an inverse microscope ZEISS Axio Observer.Z1. 3D cuts of single microglia cells (each delimited by a green box in the 2D images) with corresponding Deep Red (A) or PKH26 (B) localization are shown in the lower pictures. Respective right halves of the cells illustrate that the vesicle content was uniformly released (A), whereas the leftovers of the vesicle membranes are located at certain foci (B). 3D Visualizations were obtained using a combination of both surface and volume rendering using the computer algebra system Wolfram Mathematica 12.0 (Champaign, IL, USA) (AVI 24.1 MB)

ESM 2 (DOCX 2.29 MB)

## Data Availability

The datasets generated and analyzed during the current study are available from the corresponding author on reasonable request.
