# Supplementary material for: Extracellular Vesicles of Mesenchymal Stromal Cells Can be Taken Up by Microglial Cells and Partially Prevent the Stimulation Induced by β-amyloid
Source: Stem Cell Rev Rep. 2022 Jan 26;18(3):1113–26. doi: 10.1007/s12015-021-10261-4 (PMC8942956; doi:10.1007/s12015-021-10261-4)
Supplement: Supplementary file 2 — (DOCX 2.29 MB) [file 12015_2021_10261_MOESM2_ESM.docx]

**SUPPLEMENTARY MATERIAL AND METHODS**

**Isolation and culture of human MSCs from adipose tissue (ASCs)**

Human subcutaneous adipose tissues from different age groups and donors were obtained from a beauty Clinic in Leipzig, Germany. Approval to perform the study was obtained from the Ethical Commission (291-16-ek) of the University of Leipzig and after written informed consent from all the donors.

Human ASCs were isolated as described previously [Effimenko et al., *Journal of translational medicine*, vol.9, p.10, 2011]. Adipose tissue was cut into 1-2 mm pieces and extensively washed with phosphate-buffered saline (PBS, Thermo Fisher Scientific, US). Next, samples were centrifuged at 600 x g for 10 min. After removing the top layer of oil, the lipid phase was harvested from the top of the falcon tube and transferred into a 15 ml tube containing an equal volume of collagenase IV (final concentration: 200 U/ml; Sigma-Aldrich Chemie GmbH, Munich, Germany). The tubes were then placed in a 37°C water bath with intermittent shaking for 30 min. After the incubation time, an equal volume of culture media Dulbecco's modified Eagle's medium, 1 g/L D-glucose, and GlutaMAX^TM^ (DMEM-LG, Life Technologies) supplemented with 20% fetal bovine serum (FBS, Life Technologies) was added and centrifuged at 600 x g for 5 min. As result of the digestion, three layers are visible after centrifugation. The first layer contains the adipocytes, the second layer consists of enzymes, and the third layer includes the stromal vascular fraction (SVF), which consists of stem cells and endothelial cells. The first and second layers were carefully removed with a pipette and 10 ml of culture media was added to the layer containing the SVF. The SVF was sieved using a 40 μm nylon mesh (Falcon® 40 µm Cell Strainer, Life Sciences) into a 50 ml tube. The filtered supernatant, which contains ASCs, was centrifuged at 600 x g for 5 min. The supernatant was removed and 15 ml of growth media was added and transferred into a T75 flask.

**Differentiation assays for human adipose stromal cells**

***Chondrogenic differentiation***

Passage 2–5 ASCs were plated in 6-well plates at a density of 5x10^4^ cells per cm^2^. Chondrogenesis was induced with medium containing DMEM, 4.5 g/L D-glucose (DMEM-HG GlutaMAX™, Life Technologies), supplemented with 10% FBS, 1% Insulin Transferrin Selenium (ITS) (Sigma-Aldrich), 10^−8^ M dexamethasone (Sigma-Aldrich), 50 µg/ml ascorbic acid-2-phosphate, 10 ng/ml transforming growth factor-β1 (TGF-β1) (Peprotech, Hamburg, Germany) and 20 µM linoleic acid (Sigma-Aldrich). The medium was replaced every three days, for 14 days. The same media with only FBS added was used as negative control. After 14 days, the cells were washed two times with PBS and fixed with 10% buffered Formalin-Solution for 2 h. The extracellular matrix, rich in sulphate glycosaminoglycan, was visualized using an Alcian blue staining [Naaldijk et al. BMC Biotechnology 2012, 12:49].

***Adipogenic differentiation***

ASCs were seeded at 1x10^4^ cells/cm^2^ into 24-well plates and cultured in medium containing DMEM-High Glucose (HG) with GlutaMAX with 10% FBS, 0.1 mM indomethacin (Sigma-Aldrich), 0.5 mM 3-isobutyl-1-methylxanthine (Sigma-Aldrich), 10^−8^ M dexamethasone (Sigma-Aldrich) and 1% ITS (Sigma-Aldrich). The medium was changed twice a week. The same media with only FBS added was used as negative control. After 14 days, the medium was removed and the cells were washed twice with PBS and then fixed with 4% buffered paraformaldehyde (PFA) (Sigma-Aldrich) for 15 min. Next, the cells were washed twice with double-distilled water (ddH_2_O) and twice with 60% isopropanol before the Oil Red O solution (0.45% oil red O dissolved in 100% isopropanol) was added for 20 min. The cells were finally washed twice with PBS before being photographed.

***Osteogenic differentiation***

ASCs were plated at 5x10^3^ cells/cm^2^ into 24-well plates. Osteogenic induction medium was composed of DMEM-LG with GlutaMAX and 10% FBS supplemented with 10^−8^ M dexamethasone and 50 µg/ml ascorbic acid-2-phosphates. The medium was changed every three days during 14 days. The same media with only FBS added was used as negative control. After this period, the cells were washed twice with PBS and then fixed in 70% ethanol for 30 min. Later, the cells were washed twice with ddH2O and stained with 0.1 mg/ml naphthol ASBI (Sigma-Aldrich) plus 0.6 mg/ml Fast Red TR (Sigma-Aldrich) in 0.05 M Tris-HCl (pH 8.5) for 30 minutes. The cells were finally washed twice with ddH2O before being photographed.

**BV-2 microglia cell culture**

BV-2 cells, a murine microglia cell line, was cultured in DMEM-HG with GlutaMAX (Life Technologies) supplemented with 10% FBS (Life Technologies) at 37°C with 8% CO_2_. Subculture was done twice a week.

**β-amyloid aggregation**

To induce the formation of Aβ aggregates, 0.1 mg of HFIP-treated Aβ 1-42 (Bachem, California, USA) were dissolved in Dulbecco's PBS (DPBS, Life technologies) to a concentration of 250 µM. Formation of molecular aggregates was allowed as previously reported [Teplow, *Methods in enzymology*, vol.413, p. 20, 2006. and Kummer et al., *Neuron*, vol. 71, no. 5, p. 833, 2011] at 37°C and under constant shaking during 86 h. After aggregation, the suspension was divided in aliquots and stored at -80°C.

**RNA isolation and gene quantification by real time PCR**

The expression levels of inflammatory genes encoding for TNF-α, IL-1β, IL-6 and PTGS2 were quantified using quantitative real time polymerase chain reaction (RT-PCR). The BV-2 cells were stimulated with Aβ aggregates in presence or absence of human ASC-EVs and mouse MSC-EVs during 6 h, followed by total RNA isolation of the cells using the RNA isolation kit (Qiagen, Hilden, Germany) according to the manufacturer’s instructions. Potential DNA contaminations were removed by treating the samples with a DNA digestion reaction (DNA-free kit, Thermo Scientific, Schwerte, Germany). RNA integrity was assessed using a total RNA assay for eukaryote cells in a Nano Photometer (Agilent Technologies, Santa Clara, USA). Reverse transcription was done using the RevertAid first strand cDNA reverse synthesis kit (Thermo Scientific). Quantitative RT-PCR was performed using a SYBR green Master Mix (Promega) and the primer mix required to amplify the respective sequences as reported previously [Jaimes et al., *Stem cells*, vol. 35, no. 3, p. 812, 2017]. As endogenous control glyceraldehyde-3-phosphate dehydrogenase (GAPDH) was used. The relative gene expression was calculated using the 2^-ΔΔCt^ method.

**Enzyme linked immunofluorescent assay (ELISA) to evaluate TNF-α secretion**

BV-2 cells were incubated with Aβ aggregates, human ASC-EVs or mouse MSC-EVs during 24 h. Cell culture supernatants were evaluated for TNF-α secretion by ELISA using the antibody pairs from BD Pharmingen (coating antibody clone: TN3-19.12, detection antibody clone: 516D1A1).

**Assessment of nitric oxide secretion**

Nitrite (NO (2)-) concentration was evaluated in the cell culture supernatant from BV-2 cells stimulated with Aβ aggregates for 24 h in presence or absence of EVs using a Griess reagent (Sigma-Aldrich) reaction. Nitrite is one of the nonvolatile breakdown products from NO. Equal volumes of cell culture supernatant and Griess reagent were incubated during 10 min at room temperature in the dark. The colorimetric reaction was evaluated at 540 nm of absorbance. Sodium nitrite was used for the standard curve.

**Characterization of CD36 and CD206 expression**

BV-2 cells were seeded into 24‐well plates with a density of 2.1x10^5^ cells/cm^2^ in DMEM-HG with GlutaMAX and 2% FBS. Next day, BV-2 cells were pretreated with LPS (1 µg/ml) for 3 h, washed 1 time, followed by a 24 h incubation, with or without Aβ aggregates (10 μM) and with or without mouse MSC-EVs or human ASC-EVs to a density of 6.5x10^4^ ± 1.5x10^4^ EVs/cell (NTA quantification). After incubation, cells were stained with antibodies (BD Pharmingen, Heidelberg, Germany) specific for the cell surface markers CD36 (PE, clone CRF D-2712) and CD206 (APC, clone C068C2). The cells were acquired on a FACS Canto II flow cytometer (BD Biosciences), and the data were analyzed using the software BD FACS Diva (BD Biosciences).

**Phagocytosis of latex beads, BV-2 *in vitro* assay**

BV-2 cells were seeded into 24‐well plates with a density of 2.1x10^5^ cells/cm^2^ in DMEM-HG with GlutaMAX and 2% FBS. Next day, BV-2 cells were pretreated with LPS (1 µg/ml) for 3 h followed by a 24 h incubation, with or without Aβ aggregates (10 μM) and with or without mouse MSC-EVs or human ASC-EVs to a density of 6.5x10^4^ ± 1.5x10^4^ EVs/cell (NTA quantification) (stimulation phase). To evaluate the general phagocytic capacity of the BV-2 cells, the media was replaced by media with fluorescently labeled carboxylate-modified polystyrene latex beads (10 beads/cell) (Sigma-Aldrich) added for 3 h (phagocytic phase). Cells were washed with PBS, trypsinized for 15 min, collected in tubes and centrifuged at 125 x g for 8 min. Cell pellet was resuspended in 0.4 ml PBS and cells were acquired in the Alexa Fluor® 488 channel by FACS Canto II (BD Biosciences). The percentage of Alexa Fluor® 488-positive cells was determined on the single‐cell population using the BD FACS Diva software.

**Extracellular vesicles fluorescent labelling for microscopic imaging**

To visualize/detect the phagocytosis of human ASC-EVs, BV-2 cells were cultured in 35 mm µ-Dish with ibiTreated bottom (ibidi, Martinsried, Germany) during 4 days (seeding rate: 1.5x10^3^ cells/cm²). On day 4, the EVs were freshly stained with either PKH26 Red Fluorescent Cell Linker (Sigma-Aldrich) or CellTracker™ Deep Red dye (Thermo Fisher Scientific, Waltham, MA, USA) according to the manufacturer’s protocols. Briefly, after ultra-centrifugation step at 70,000 x g for 30 min, approximately 3x10^10^ EVs were stained either with PKH26 Red Fluorescent Dye Solution (2x10^-6^ M) resuspended in Diluent C with periodic mixing for 5 min followed by adding and incubation with an equal volume of FBS for 1 min to stop the staining reaction or with pre-warmed CellTracker™ Deep Red Dye Solution (15 µM) for 45 min at 37°C. After another two ultra-centrifugation steps, EVs were resuspended in HBSS media and added to the microglia cells. The labelled human ASC-EVs and BV-2 cells were co-cultured for up to 5 days.

**SUPPLEMENTARY FIGURE LEGENDS**

**Supplementary Figure 1. Extracellular vesicle isolation.**

The EVs were isolated using differential (ultra)-centrifugation steps. Initially human ASC or mouse MSC culture supernatants were centrifuged at 500 x g for 10 min to remove cells and cell debris. To deplete large vesicles, samples were centrifuged again at 10,000 x g during 30 min and the final concentration of EVs was achieved by centrifuging the supernatant at 70,000 x g during 90 min. All centrifugation steps were performed at 4°C. EV pellets were resuspended in 0.9% NaCl or BV-2 cell culture medium and stored at -80°C, depending on the application.

**Supplementary Figure 2. Overview of the experimental procedure.**

Amyloid-β aggregation (1-42) was performed by incubation of Aβ for 86 h at 37°C in PBS. For stimulation experiments, BV-2 cells were primed by LPS followed by Aβ stimulation in the presence and absence of EVs.

**Supplementary Figure 3. Stimulation with different concentrations of Aβ aggregates.**

Resting BV-2 cells were primed for 3 h with 1 µg/ml LPS before stimulation with 1 µM, 2.5 µM, 5 µM, 10 µM and 20 µM of Aβ (1-42) aggregates for 24 h. The cell culture supernatants were finally analyzed for secretion of **(A)** TNF-α by ELISA and **(B)** NO using a Griess reagent.

**Supplementary Figure 4. Latex bead phagocytosis assay**.

EVs from the human ASC and mouse MSC supernatants were isolated via differential (ultra)-centrifugation. Resting BV-2 cells were primed for 3 h with 1 µg/ml LPS before 24 h stimulation with 10 µM of Aβ (1-42) aggregates in the presence and absence of 6.5x10^4^ ± 1.5x10^4^ EVs/cell (NTA quantification). Afterwards BV-2 cells were co-cultured with fluorescently labeled carboxylate-modified polystyrene latex beads (10 beads/cell) for 3 h at 37°C. As an internal phagocytosis control, LPS primed cells were incubated with beads for 3 h at 4°C. Cells were analyzed by flow cytometry to evaluate fluorescently positive and negative cells (n=3).

**SUPPLEMENTARY VIDEO LEGEND**

After labeling ASC-EVs with CellTracker™ Deep Red dye, which labels the membrane, EVs were co-cultured with BV-2 cells. Live cell imaging was performed using an inverse microscope ZEISS Axio Observer.Z1 equipped with AxioCam MR Rev3 camera, 63x/1.40 oil objective and incubation setup at 37 °C and 8% CO_2_. Brightfield images of 80 ms exposure time and fluorescence images of 220 ms exposure time were taken every 2 seconds for a timeframe of 240 seconds. The video is accelerated 10 times from 0.5 to 5.0 frames per second (fps).

**SUPPLEMENTARY FIGURES**

**Supplementary Figure 1**


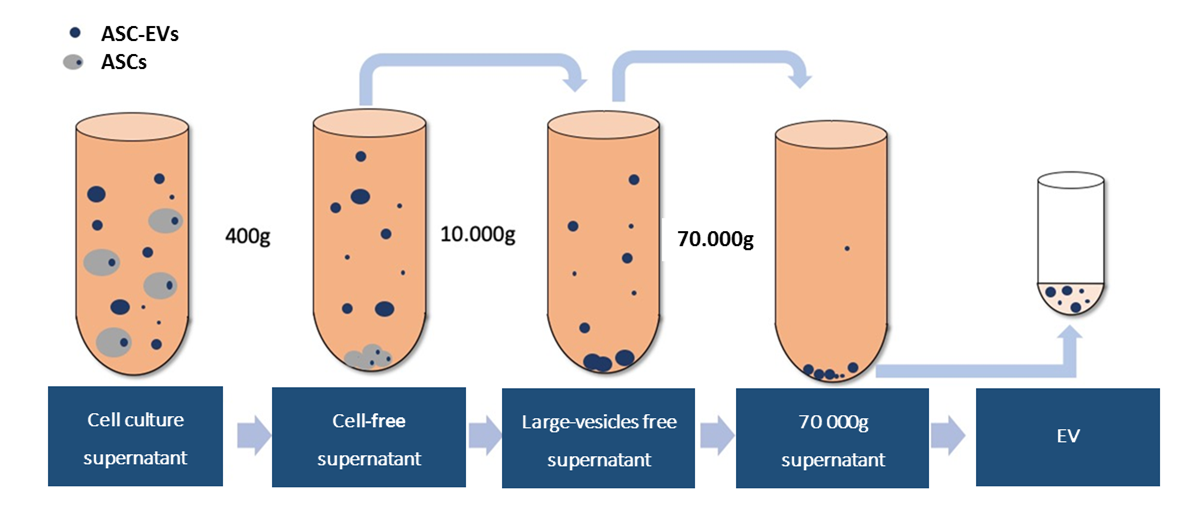


v

70,000 x g

supernatant

v

v

v

Large vesicles

free supernatant

Cell-free

supernatant

Cell culture

supernatant

EVs

**400 x g**

**10,000 x g**

**70,000 x g**

**ASC EVs**

**ASCs**

**Supplementary Figure 2**


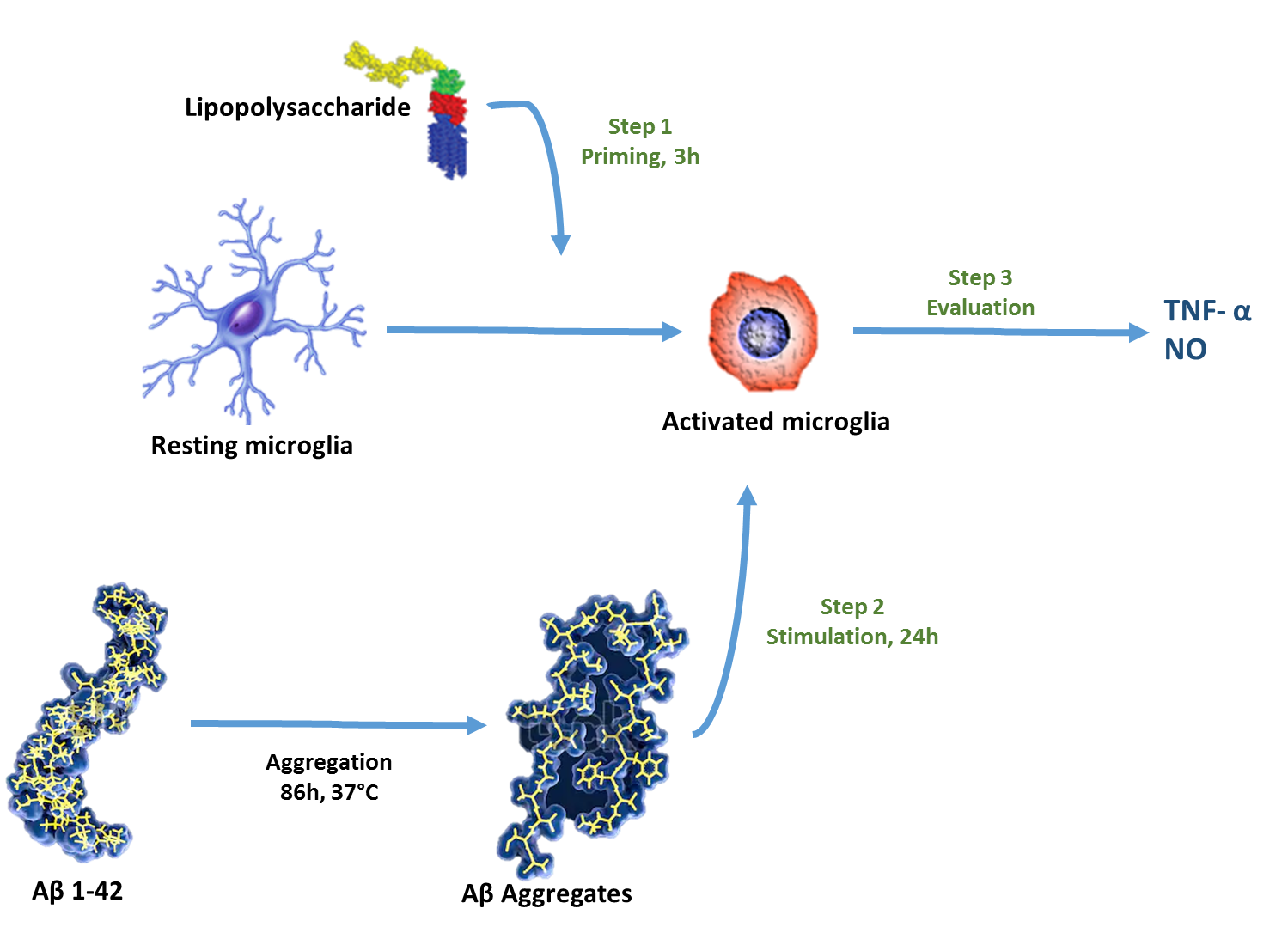

**Supplementary Figure 3**

**A**

**B**

**Supplementary Figure 4**

**Bead phagocytosis**

**-**
